# Different replication behavior of a contemporary porcine hemagglutinating encephalomyelitis virus strain Gent/PS412 compared with the historical neurotropic reference strain VW572

**DOI:** 10.1186/s13567-026-01767-1

**Published:** 2026-06-24

**Authors:** Ateeqa Aslam, Waqar Saleem, Ines Zarak, Jolien Van Cleemput, Hans Nauwynck

**Affiliations:** Laboratory of Virology, Department of Translational Physiology, Infectiology and Public Health, Faculty of Veterinary Medicine, Ghent University, Merelbeke, 9820 Ghent, Belgium

**Keywords:** Porcine hemagglutinating encephalomyelitis virus, PHEV, virus tropism, virus replication, virus strain-dependent pathogenesis, RPD, nasal mucosa explant, ethmoidal mucosa explant, PoRECs, coronavirus spike protein

## Abstract

Porcine hemagglutinating encephalomyelitis virus (PHEV), first identified in 1957, has been described as a neurotropic virus of pigs. However, more recent strains were mainly associated with respiratory problems. This study compared the replication kinetics of the historical neurotropic PHEV strain isolated in Belgium in 1972 (VW572) with the replication kinetics of a contemporary 2020 strain (PS412; isolated from nasal secretions during a monitoring study), using RPD (rein de porc diploid) cells, nasal and ethmoidal mucosa explants, and primary porcine respiratory epithelial cells (PoRECs). EGTA was used to disrupt intercellular junctions to facilitate the access of the virus to basolateral receptors. In RPD cells, VW572 showed an early advantage in initiating infection, whereas PS412 exhibited stronger replication and viral release at later stages, highlighting distinct replication dynamics between the two strains. The replication kinetics of both strains in nasal explants and PoRECs did not differ significantly. In ethmoidal explants, PHEV only replicated in sustentacular cells with VW572 infecting more cells than PS412. EGTA treatment had no effect on the PHEV infection in nasal explants but significantly increased in PoRECs and ethmoidal explants for VW572, suggesting a stronger reliance on basolateral receptors in sustentacular cells. Genomic analysis identified 2 spike protein mutations that led to amino acid changes at the receptor binding site: N512K, K576E. These changes switched the regional polarity on the surface of the spike protein that may affect the PHEV receptor use. In conclusion, the historical and contemporary PHEV isolates VW572 and PS412 replicate differently in the respiratory/neural tissues of the upper respiratory tract which may be associated with amino acid variations in the receptor binding domain of the spike protein.

**Supplementary Information:**

The online version contains supplementary material available at 10.1186/s13567-026-01767-1.

## Introduction

Porcine hemagglutinating encephalomyelitis virus (PHEV) is a member of the *Coronaviridae* family, classified under the *Betacoronavirus* genus and *Embecovirus* subgenus [1]. It is an enveloped, positive sense, single-stranded RNA virus with a genome of approximately 25–30 kbps in length [1, 2], organized into a series of multiple open reading frames (ORFs) that encode four essential structural proteins, including spike (S), envelope (E), membrane (M) and nucleocapsid (N), like all other coronaviruses [1, 3]. The S protein, consisting of two subunits (S_1_ and S_2_), plays a vital role in viral entry in the host cell and pathogenesis by mediating receptor binding (S_1_) and membrane fusion (S_2_) [4, 5]. Additionally, the genome of PHEV and some other betacoronaviruses encode an additional structural protein called haemagglutinin esterase (HE), which forms shorter spikes on the viral surface and is known to interact with host glycans [6]. Genomic analysis has revealed sequence variations among different PHEV strains, particularly within the S protein, which may play a role in host adaptation and immune evasion [7].

PHEV emerged in 1957 in Ontario, Canada, following an outbreak in piglets characterized by severe clinical signs including repeated vomiting, retching, anorexia and emaciation [8, 9]. Subsequent cases of viral encephalomyelitis in the piglets of this region were reported in 1959 and 1962 [1, 10]. In 1972, similar cases of viral encephalomyelitis were observed in Belgium [11, 12]. The outbreaks of PHEV continued to be documented in other swine-producing countries like China (1985), Republic of China (ROC) (1994) and Argentina (2006) [6, 13–15]. Serological and epidemiological surveys indicate that PHEV is widespread among pig populations [1, 16]. This virus primarily infects upper respiratory mucosa, tonsils and lungs [1], followed by a spread to the central nervous system [17].

PHEV uses cell-surface glycans such as sialic acid and heparan sulfate as attachment factors [18, 19]. In neuronal cells, it uses neuronal cell adhesion molecules (NCAM) as receptors for propagation in neural cells [20]. CD81 has been identified as a receptor that PHEV uses to infect murine neuronal cells, highlighting a mechanism of neurotropism in mice [21]. Dipeptidyl peptidase 4 (DPP4, CD26) has been shown to serve as a coreceptor or cofactor for PHEV infection, facilitating viral attachment into the respiratory cells [7]. More recently, dipeptidase 1 (DPP1) has been identified as a potential functional receptor for PHEV, mediating spike-receptor interactions and viral entry [22]. It enters its host cell through clathrin-mediated endocytosis [19]. The historical VW572 strain, isolated in 1972 from tonsils of 2 pigs in Belgium, has served as a reference strain for PHEV studies [11]. Currently, vomiting and wasting disease and encephalomyelitis associated with PHEV infections are no longer reported. This could be due to the general passive immunity in piglets that protects them or due to an adaptation of the virus over time. Comparative studies of PHEV strains are crucial for elucidating potential differences in viral replication kinetics and pathogenicity. Such analyses can provide valuable insights into viral evolution and may inform strategies for disease control and prevention. Previous research has demonstrated that factors such as virus strain, age at infection, replication dynamics and spread influence the clinical presentation of PHEV in pigs [17, 23].

In this study, we compared the replication kinetics of a recently isolated Belgian PHEV strain from 2020 (PS412; isolated from nasal secretions during a monitoring study) with the historical Belgian VW572 strain. This was accomplished by analyzing viral growth curves in RPD cells, nasal and ethmoidal mucosa explants and porcine respiratory epithelial cell cultures. It was aimed to understand tropism and replication differences between the historical and contemporary PHEV strain and to better elucidate its behavior in vivo. In addition, we genetically compared both strains to identify differences and to link these with the observed replication kinetics.

## Materials and methods

### Viruses and cells

Two PHEV strains (VW572 and Gent/PS412) were used in this study. VW572 is a historical strain isolated from the tonsils of two diseased pigs during a Belgian outbreak of PHEV in 1972. PS412 was isolated from nasal swabs collected from a pig farm in The Netherlands during a monitoring study in 2020. The GenBank accession numbers for VW572 is DQ011855 and for PS412 is PV820712. Stocks of both strains were prepared on rein de porc diploid (RPD) cells. RPD cells (250,000 cells/mL) were seeded on glass inserts in a 24-well plate and allowed to attain confluency in culture medium; RPMI Glutamax (Sigma-Aldrich, St. Louis, MO, USA), supplemented with 10% fetal calf serum (FCS, Sigma-Aldrich, St. Louis, MO, USA), 100 U/mL penicillin (ThermoFisher Scientific, Paisley, UK), 0.1 mg/mL streptomycin (ThermoFisher Scientific, Paisley, UK) and 0.1 mg/mL gentamicin (ThermoFisher Scientific, Paisley, UK).

### PHEV inoculation

RPD cells were inoculated with PHEV VW572 or PS412 at a multiplicity of infection (MOI) of 0.5 (200 µL each) for 1 h at 37 °C. After the inoculation, the cells were washed 3 times with phosphate-buffered saline (PBS), supplemented with calcium and magnesium. After washing, 1 mL of culture medium was added, and the cells were further incubated at 37 °C and 5% CO_2_. The RPD cells were fixed at room temperature (RT) with 400 µL of 4% paraformaldehyde for 20 min after 0, 3, 6, 9, 12, 18, 24, and 48 h post-inoculation (hpi). Non-infected RPD cells were also fixed and used as negative control.

### Determination of extracellular and intracellular virus titers

For the determination of the extracellular titer, the culture medium was collected from each well at 0, 1, 3, 6, 9, 12, 18, 24, and 48 hpi. RPD cells were seeded in 96-well plates and incubated for three days to allow the formation of a confluent monolayer. Subsequently, 300 µL of the collected supernatant was used to prepare 10-fold serial dilutions, which were added to the cells for virus titration. For the determination of the intracellular titer, the monolayer of infected RPD cells was removed from the inserts using a cell scraper and collected in Eppendorf tubes. Cells were freeze-thawed twice. Subsequently, the tubes were centrifuged at 13 000 rpm for 10 min to pellet the cell debris, and the virus-containing supernatant was collected and used for intracellular virus titration using the same procedure. After three days of incubation, the cells were fixed and viral antigen was detected by immunoperoxidase monolayer assay (IPMA) staining. The virus titer was calculated as 50% tissue culture infectious dose (TCID₅₀) using the Reed and Muench method [24].

### Porcine respiratory mucosa explants

Three piglets (3 weeks old, Belgian Piétrain) were euthanized using pentobarbital at 12.5 mg/kg body weight intravenously according to the ethical guidelines of the Faculty of Veterinary Medicine, Ghent University. The seronegative status of the pigs was ensured by first testing the blood from the sows, and then from the euthanized pigs themselves, through a virus neutralization (VN) test. The starting dilution was 1/2. An antibody titer less than 2 was considered seronegative. The nasal mucosa was stripped carefully from the nasal septum with the help of a sterile surgical blade and tweezers. The ethmoid turbinates were identified and carefully collected to keep the entire ethmoid structure intact. The olfactory mucosa was gently separated from the bone by using a sterile surgical blade. The nasal mucosa and olfactory mucosa were placed in separate petri dishes, containing transport medium [PBS with calcium and magnesium, supplemented with 100 U/mL penicillin, 0.1 mg/mL streptomycin, 0.1 mg/mL gentamicin and 0.25 µg/mL amphotericin B (ThermoFisher Scientific)], with the epithelial side facing upwards.

The nasal respiratory mucosa was observed under an Olympus IX50 light microscope to check the cilia beating activity. Afterwards, the mucosa was cut into 0.25 cm^2^ squares using a sharp surgical blade to make explants. The nasal explants and olfactory tissue were placed onto fine-mesh wire gauzes within 6-well plates with the epithelial side facing upwards and were cultivated at an air–liquid interphase (ALI) in serum-free media containing DMEM/RPMI in a 1:1 ratio (ThermoFisher Scientific, Paisley, UK), supplemented with 100 U/mL penicillin, 0.1 mg/mL streptomycin, 0.1 mg/mL gentamicin, and 0.25 µg/mL amphotericin B for 24 h at 37 °C and 5% CO_2_.

### Pretreatment of respiratory mucosa explants with EGTA

After 24 h of pre-incubation, the explants were shifted to 24-well plates and washed with serum-free culture medium twice to remove the mucus layer produced by the explants. They were then treated with ethylene glycol-bis (2-aminoethyl ether)-N, N, N’, N’-tetraacetic acid (EGTA) (VWR International, Leuven, Belgium) before virus inoculation to see the effect of the disruption of intercellular junctions on PHEV infection using an agarose model [25]. 3% agarose (1 mL) diluted in distilled water was placed in the required wells of 24-well plates. When the agarose layer solidified, the explants were placed on top of it with the epithelium facing upwards. Additional agarose was added on the lateral sides to block the lateral surfaces of explants. Once the explants were fixed properly, 1 mL of 8 mM EGTA in Dulbecco’s PBS without calcium and magnesium was added on top of the explants and incubated for 1 h at 37 °C and 5% CO_2_. As mock treatment, Dulbecco’s PBS without EGTA was added to one of the explants. Immediately after the EGTA treatment on the agarose model, the explants were washed. One of the EGTA-treated explants and one of the mock-treated explants were fixed in 4% paraformaldehyde for 24 h for histomorphology analysis.

### Porcine respiratory epithelial cells (PoRECs)

The nasal septum and turbinate tissues were collected from 3-week-old piglets in PBS containing calcium and magnesium, supplemented with 100 U/mL penicillin, 0.1 mg/mL streptomycin, and 0.1 mg/mL gentamicin and 0.25 µg/mL amphotericin B [26]. The nasal septa and turbinates were incubated within an enzymatic solution containing 1.12 mg/mL pronase (Roche Diagnostics, Mannheim, Germany) and 80 μg/mL DNase I (Roche Diagnostics, Mannheim, Germany) in calcium and magnesium free PBS supplemented with 25 mM glucose (VWR International, Leuven, Belgium), 1% sodium pyruvate (Gibco, Paisley, UK), 0.1 mg/mL streptomycin, 100 U/mL penicillin, and 5 μg/mL amphotericin B at 4 °C for 24 h to get PoRECs. After enzymatic digestion, the detached cells from nasal septa and turbinates were incubated in DMEM/F12 (1:1 ratio) containing 1% non-essential amino acids (NEAA) (Gibco, Paisley, UK), 0.12% Insulin-Transferrin-Selenium-Ethanolamine (ITS-X) (Gibco, Grand Island, NY, USA), 0.1 mg/mL streptomycin, 100 U/mL penicillin, and 5 μg/mL amphotericin B in a cell-culture petri dish for 4 h at 37 °C and 5% CO_2_ for adherence of fibroblasts to the surface of petri dish. Then, the number of viable isolated PoRECs were counted and seeded at a density of 800 000 cells in a transwell insert with a 1.0 μm pore size (Falcon) pre-coated with type IV collagen (Sigma-Aldrich, St. Louis, MO, USA) in a 12-well plate, and cultivated overnight in DMEM/F12 (1:1 ratio) medium, supplemented with 5% fetal calf serum (FCS) (Sigma-Aldrich, St. Louis, MO, USA), 1% NEAA, 0.1 mg/mL streptomycin, 100 U/mL penicillin, and 5 μg/mL amphotericin B. Then, the transwell inserts with PoRECs were washed with DMEM/F12 medium and replaced by the complete DMEM/F12 medium, further supplemented with 2% Ultroser G (Sartorius, Cergy, France). The medium was added to the bottom well to have an air–liquid interface, and the cells were cultivated for 3–5 days until a confluent monolayer was achieved. Confirmation of confluency was done by measuring the transepithelial electrical resistance (TEER). Identification of epithelial cells was done using mouse anti-human cytokeratin (1:100) as primary antibody and Alexa Fluor 594 as secondary antibody (1:500). The viability of the cells was determined by incubating the PoRECs monolayer with EMA solution (25 µg/mL) for 30 min on ice in the dark. Following incubation, the cells were exposed to candescent light for 10 min on ice. Subsequently, the EMA solution was removed, and the cells were fixed with 500 µL of 4% paraformaldehyde for 10 min at room temperature. After fixation, the nuclei were stained with Hoechst (10 µg/mL in PBS, Invitrogen) for 10 min. To assess cell viability, five representative fields per transwell were analyzed. In each field, the total number of epithelial cells was manually counted under a fluorescence microscope using a 40× objective lens and a 10× eyepiece (total magnification: 400×). EMA positive cells were identified as non-viable, while EMA negative cells were considered viable. The percentage of viable cells was calculated as the proportion of EMA negative cells out of the total number of cells per field, and a 95% confidence interval was computed across the five fields to ensure statistical reliability.

### PHEV inoculation

The explants in agarose were inoculated with 1 mL of PHEV at a titer of 10^6^ TCID_50_/mL suspended in serum-free DMEM/RPMI 1640 medium for 1 h at 37 °C and 5% CO_2_. After incubation, the explants were washed with serum-free medium three times and transferred to their gauzes and incubated in serum-free DMEM/RPMI 1640 medium in an air–liquid interface. After 0, 24, and 48 hpi, the explants were embedded in methylcellulose and stored at −70 °C until further processing.

For PHEV inoculation on PoRECs, DMEM/F12 medium (supplemented with 2% Ultroser G, Sartorius, Cergy, France), was removed, and 200 μL of PHEV at a titer of 10^6^ TCID_50_/mL were added either via apical route, with and without EGTA treatment, or the basolateral surface for 1 h at 37 °C and 5% CO_2_. For the apical route inoculation with EGTA, cells were pre-treated with 25 mM EGTA in Dulbecco’s PBS (VWR International, Leuven, Belgium) for 30 min at 37 °C and 5% CO_2_. After washing with medium, cells were further incubated and fixed at 0, 24 and 48 hpi with absolute methanol at −20 °C for 20 min. Fixed cells were stored at −20 °C until further processing. At each time point, supernatants were harvested from the explant cultures and PoRECs and used for virus titration on RPD cells to check extracellular virus titers. The virus titer was calculated as the 50% tissue culture infectious dose (TCID₅₀) using the Reed and Muench method.

### Immunofluorescence staining

Immunofluorescence staining was performed to identify PHEV-infected cells in explants and PoRECs. Cryosections of 10 µm thickness were cut from each explant and loaded onto 3-aminopropyltriethoxysilane-coated (Sigma-Aldrich, St. Louis, MO, USA) glass slides using the cryostat for the nasal and olfactory explants. The cryosections were fixed in absolute methanol at −20 °C for 20 min.

To assess apoptosis in the explants, terminal deoxynucleotidyl transferase mediated dUTP nick end labelling (TUNEL) staining (Roche Diagnostics, Mannheim, Germany) was performed according to the manufacturer’s instructions. TUNEL positive cells were counted and expressed as a percentage of the total epithelial cells to quantify the extent of apoptosis.

To identify PHEV-infected cells, the cryosections and PoRECs were incubated first with avidin/biotin as blocking agent and next with biotinylated polyclonal porcine anti-PHEV antibody as primary antibody (IgG was purified and subsequently biotinylated in the laboratory) (dilution 1:20) for 1 h at 37 °C [21]. The sections were washed three times with PBS. Afterwards, the sections were incubated with streptavidin-FITC^®^ (ThermoFisher Scientific) (dilution 1:200) for 1 h at 37 °C. For the confirmation of epithelial cell, mouse anti-human cytokeratin marker (Abcam) (1:100) was used as primary antibody followed by Alexa Flour 594 conjugated goat anti-mouse IgG (1:500, Invitrogen) as secondary antibody. For identification of sustentacular cells, cytokeratin 18 polyclonal antibody (ThermoFisher Scientific) (dilution 1:400) was used as primary antibody and Texas Red conjugated goat anti-rabbit IgG (1:100) as secondary antibody on ethmoid cryosections. Hoechst 33342 (ThermoFisher Scientific) (dilution 1:100) was used to counterstain the nuclei. Finally, the slides were mounted with a glycerol mounting medium containing DABCO and analyzed with a Leica DMRBE fluorescent microscope. For PoRECs and RPD cells, five random fields per transwell and per insert were selected and cells were counted under a 40× objective lens with a 10× eyepiece (total magnification: 400×). The total number of cells and infected cells were recorded in each field, and the infection rate was calculated as a percentage. For explants, in each section, all infected epithelial cells were counted throughout the entire length of the epithelial surface. Cell counting was performed under a 40× objective lens with a 10× eyepiece (total magnification: 400×). The number of infected cells was recorded per section, and results were presented as the total number of infected cells across 10 sections. Cells in the lamina propria were excluded from the analysis to ensure specificity to the epithelial compartment. Fluorescent microscopy was done using Leica Microsystems DMRBE.

### Comparative genomic analysis

The complete genome of PHEV strain isolated in 2020 was sequenced by nanopore based sequencing using a previously described method (NCBI accession no. PV820712) [27]. The spike protein amino acid sequences of the latter strain as well as that of the classical strain VW572 (NCBI accession no. DQ011855.1) were aligned using Clustal Omega to identify sequence differences. These sequences were then modeled using AlphaFold 3, which is capable of modeling glycosylated proteins and protein complexes [28]. Structural models were visualized using ChimeraX, and amino acid substitutions were mapped onto the 3D structures to assess their spatial distribution and potential conformational impact.

### Statistical analysis

All data were expressed as mean ± standard deviation (SD) from three independent experiments with three different animals. Statistical analysis was performed using IBM SPSS Statistics (Statistica v29, TIBCO Software). Descriptive statistics were first calculated for all experimental groups. Homogeneity of variance was evaluated using Levene’s test and normality of residuals was examined with the Shapiro–Wilk test. When there was interaction between two factors, simple effects analyses were conducted. Differences between different conditions were analyzed using a one-way analysis of variance (ANOVA) followed by Tukey’s post-hoc tests and *p* < 0.05 was considered statistically significant.

## Results

### In RPD cells, VW572 is more efficient early in infection, while PS412 dominates in later stages

**Figure 1:**
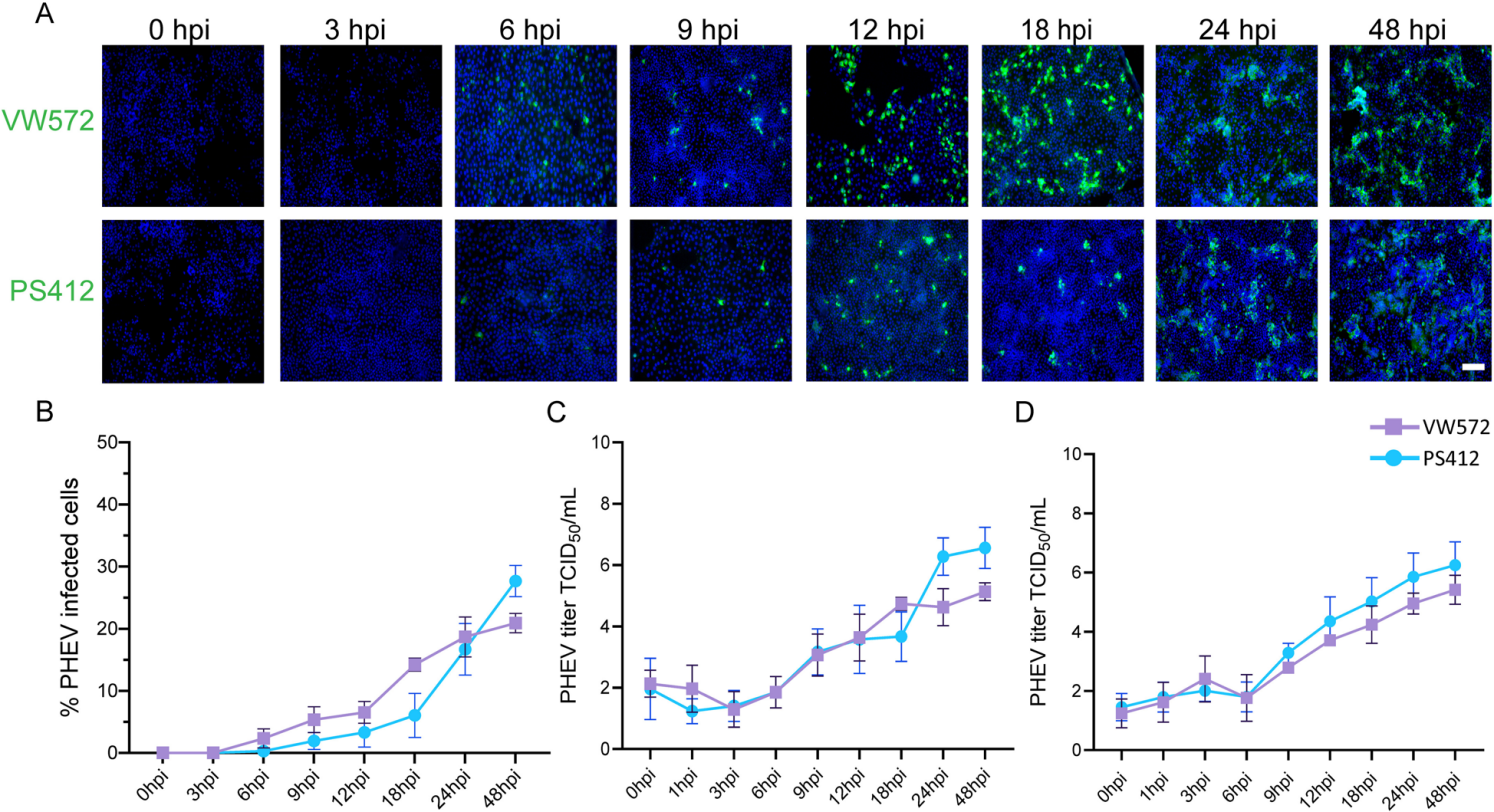
**Replication kinetics of PHEV VW572 and PS412 in RPD cells.** **A** Representative images of infected RPD cells at all collected time points. PHEV-infected cells are green; nuclei are blue. Scale bar = 50 µm **B** Percentage of infected cells for both strains. **C** Intracellular virus titers. **D** Extracellular virus titers. Data is represented as mean ± SD of 3 independent experiments. Statistical analysis was performed using a general linear model (univariate); a significant difference observed in percentage of infected cells at 18 hpi (df = 1, F = 14.580) and 48 hpi (df = 1, F = 15.675); intracellular virus titer is different at 24 hpi (df = 1, F = 11.108) and 48 hpi (df = 1, F = 11.446); extracellular titer (df = 1, F = 6.807).

To compare the difference in replication dynamics of the historical VW572 and contemporary PS412 strain of PHEV, both strains were evaluated in RPD cells. Both strains replicated well in RPD cells (Figure 1). The first virus-positive cells were observed at 6 hpi which increased over time for both strains. Immunofluorescence staining revealed time-dependent differences in the percentage of infected cells between the two PHEV strains. At 18 hpi, infection with the historical VW572 strain resulted in a significantly higher proportion of positive cells compared to the contemporary PS412 strain (*p* = 0.019). However, at 48 hpi, the percentage of infected cells was significantly higher for PS412 (*p* = 0.017). To further characterize the differences in replication dynamics between two PHEV strains, extracellular and intracellular virus titers were quantified. Analysis of extracellular virus production showed consistently and significantly higher titers for PS412 across all examined time points when compared to VW572 (*p* = 0.013). PS412 reached significantly higher intracellular titers than VW572 only at 24 hpi (*p* = 0.029) and 48 hpi (*p* = 0.028). These findings suggest that while VW572 initiates infection more efficiently at earlier stages, PS412 demonstrates enhanced replication and release at later stages of the infection cycle.

### Evaluation of apoptosis in PHEV-infected respiratory mucosa explants 

**Figure 2:**
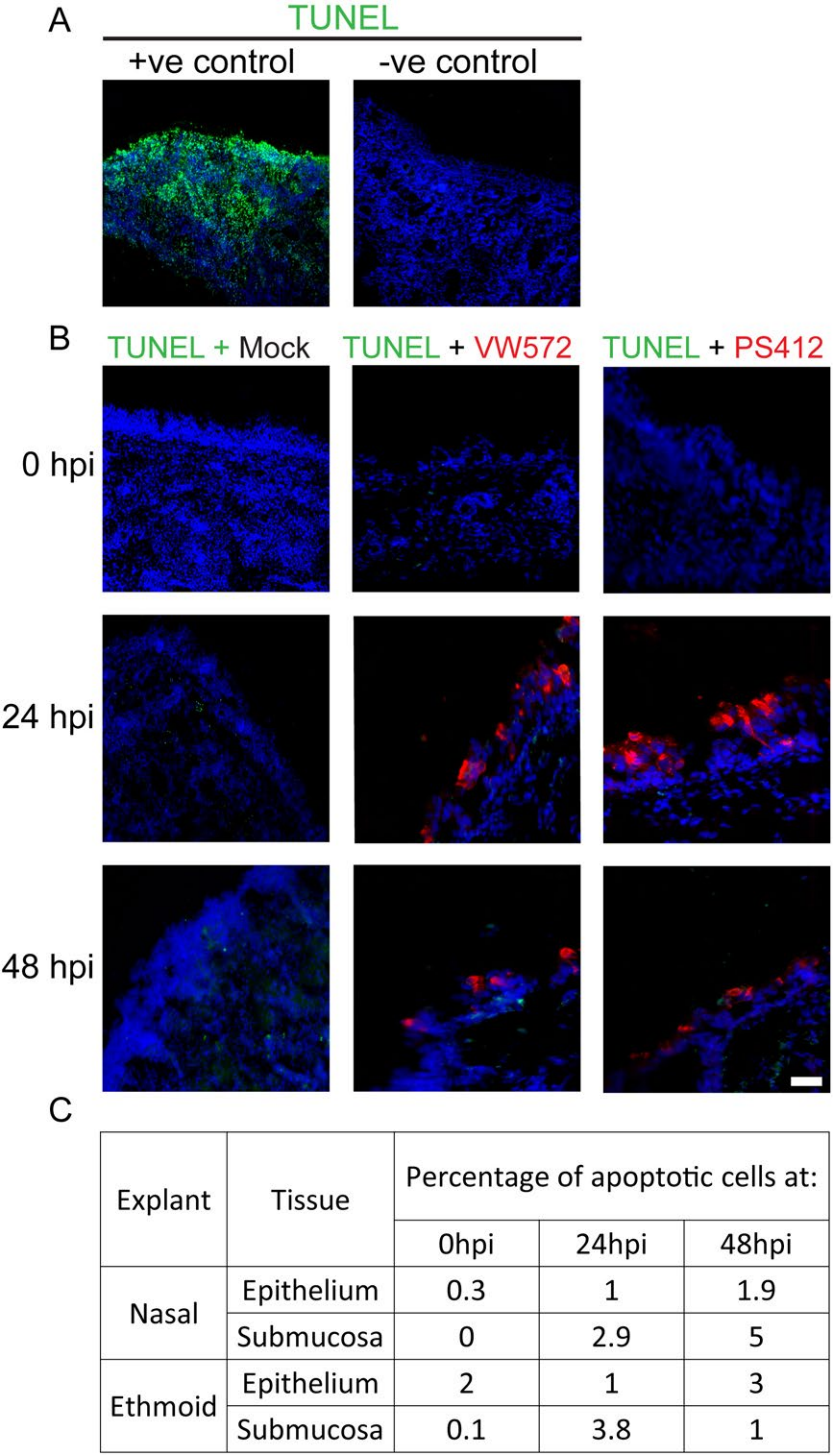
**Viability of respiratory mucosa explants upon PHEV- and mock-inoculation.** **A** Representative images of TUNEL staining of explants. Apoptotic cells are green; nuclei are blue. Scale bar = 50 µm **B** Representative images of TUNEL-positive (green) and virus infected (red) cells at different time points; nuclei are blue. **C** Average percentage of apoptotic of nasal and ethmoid explants at different time points after mock- and PHEV-inoculation.

To determine whether PHEV infection is associated with apoptosis in respiratory mucosa explants, TUNEL staining was performed to identify apoptotic cells, which appear green (Figure 2A). We assessed the viability of both mock and PHEV inoculated explants, and representative images of TUNEL positive and virus infected cells in nasal explants are shown in Figure 2B. Across all time points, the overall viability of both mucosa and submucosa remained high. Virus infected cells (red) were consistently TUNEL (green) negative, indicating a lack of detectable co-localization between viral antigen and apoptotic markers. TUNEL-positive cells were observed at low frequency in both mock and infected explants. A slight increase in TUNEL-positive cells were noted at 48 hpi, however this was comparable between conditions and is likely related to time-dependent change in ex vivo tissue culture rather than a direct virus-specific effect. Overall, PHEV infection was not associated with a marked increase in apoptosis or a reduction in explant viability under the experimental conditions.

### PHEV strains prefer apical route for nasal mucosa replication

**Figure 3:**
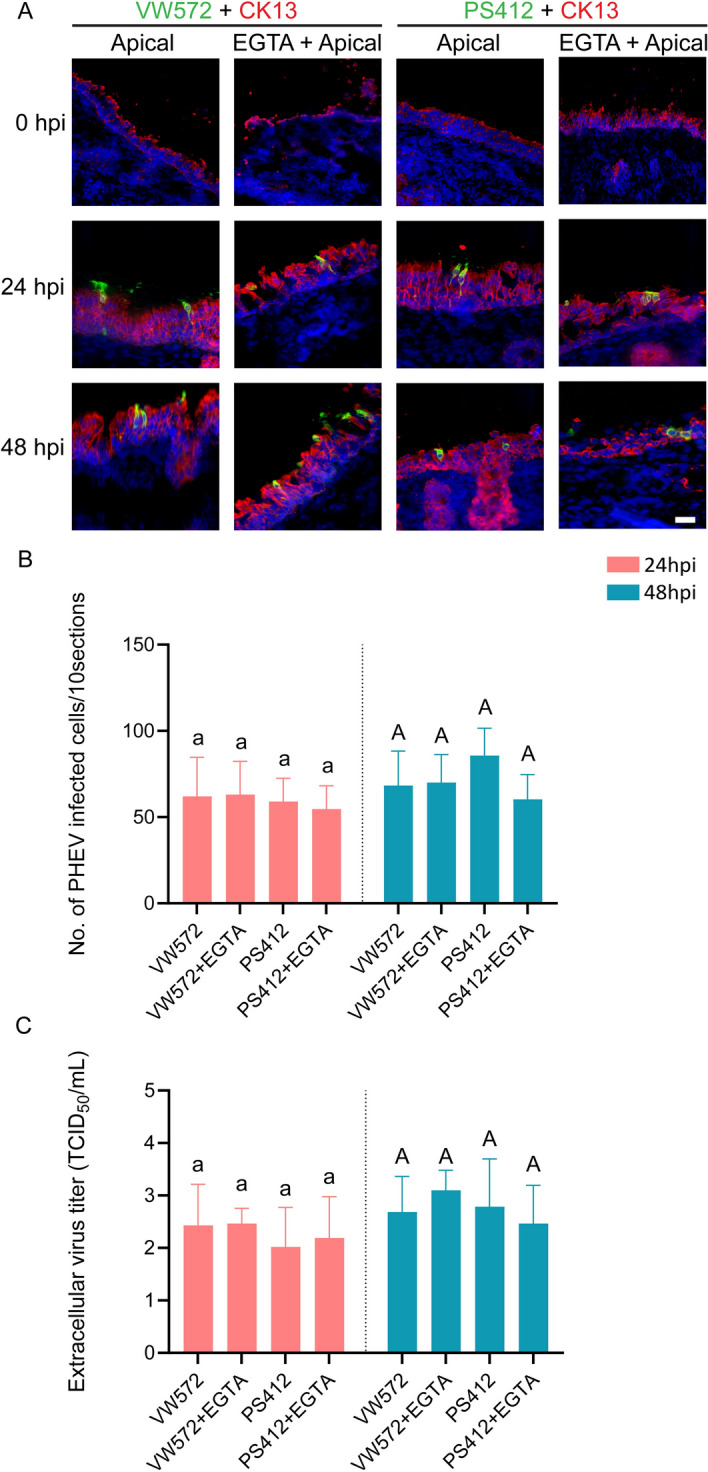
**PHEV VW572 and PS412 replication in nasal mucosa explants (n = 3).** **A** Representative images of PHEV infected cells in nasal mucosa explants at all collected time points. PHEV-infected cells are green; nuclei are blue; cytokeratin is red. Scale bar = 50 µm **B** Number of infected cells per 10 sections (magnification 400×) for both strains. **C** Extracellular virus titer for both strains. Values represent mean ± SD of three independent experiments. Significant differences between the different conditions (One-way ANOVA, df = 3, F = 0.134 at 24 hpi; df = 3, F = 1.196 at 48 hpi followed by Tukey’s post-hoc tests) are visualized using Compact Letter Display (CLD). Significant differences are indicated by letter annotations, where lowercase letters correspond to 24 hpi and uppercase letters correspond to 48 hpi. Groups not sharing the same letter are significantly different (*p* < 0.05).

Considering the crucial role of the nasal mucosa as key portal of entry for respiratory coronaviruses, difference in replication route of both PHEV strains was investigated in porcine nasal mucosa explants with a focus on apical versus basolateral infection. PHEV replication in nasal explants had a similar pattern for both strains (Figure 3A). All infected cells were epithelial cells and were present at 24 hpi and the number only slightly increased at 48 hpi for strain PS412; no significant differences were observed between the two strains (Figure 3B, *p* > 0.05). The treatment of explants with EGTA prior to infection did not significantly alter the infection patterns of both strains (Figure 3B, *p* > 0.05). This suggests that the tight junction disruption induced by EGTA treatment did not preferentially enhance the infectivity of both PHEV strains. At 24 hpi, the extracellular virus titer was 10^2.43^ TCID_50_/mL for VW572 and 10^2.02^ TCID_50_/mL for PS412. The virus titer slightly increased between 24 and 48 hpi. No significant differences were found between both PHEV strains (Figure 3C, *p* > 0.05). Overall, both PHEV strains primarily replicated via the apical route in porcine nasal mucosa, though receptor-specific studies are needed to confirm exclusive usage of the apical route.

### In olfactory mucosa, PHEV infection is restricted to sustentacular cells with strain variability

**Figure 4:**
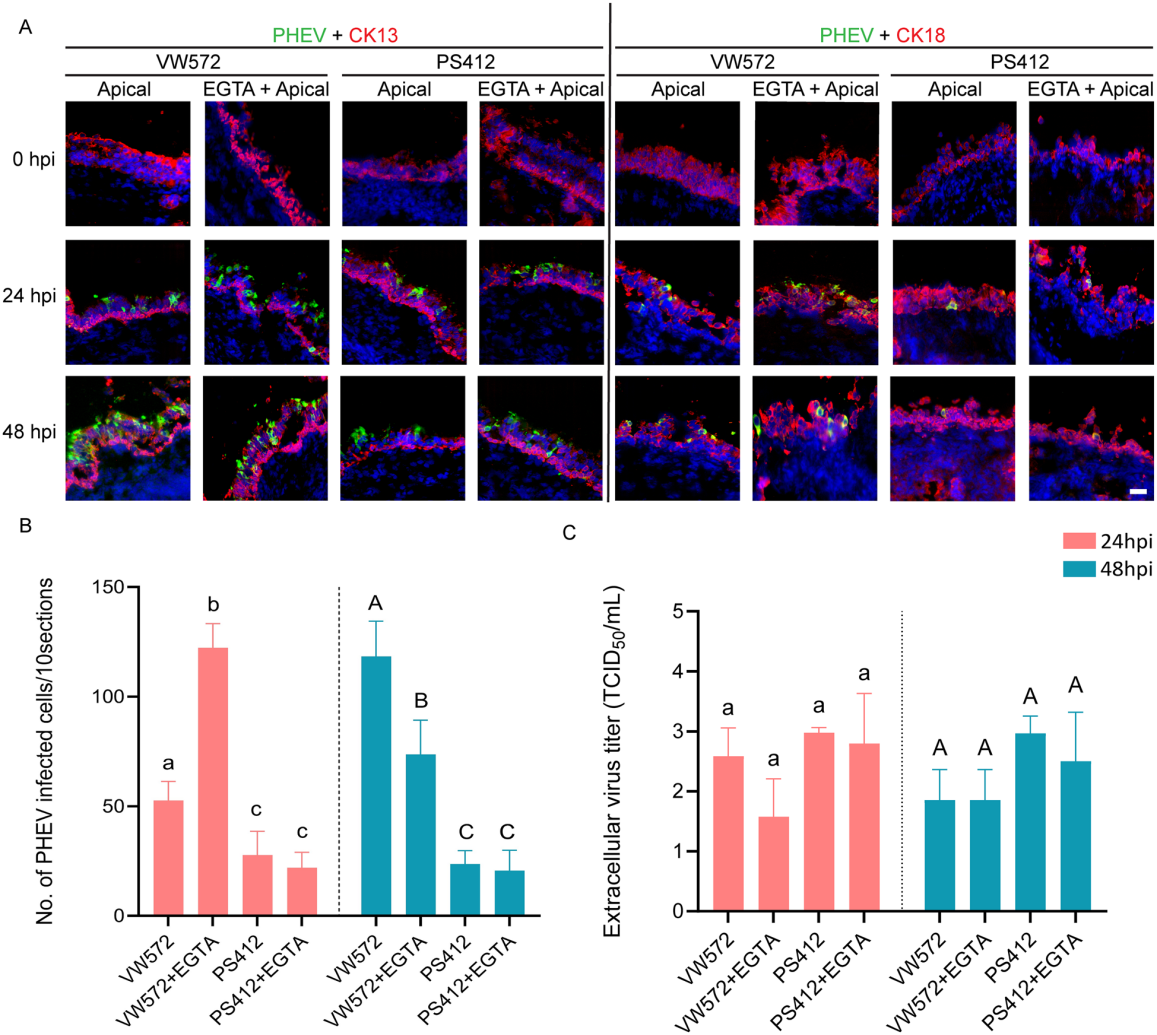
**PHEV VW572 and PS412 replication in olfactory mucosa explants (n = 3).** **A** Representative images of PHEV infected cells in olfactory mucosa explants at all collected time points. Left panels show PHEV infection in the olfactory epithelium, where PHEV infected cells are shown in green, nuclei in blue, and cytokeratin 13 (CK13) positive cells in red. PHEV infected cells are not double positive for CK13. Right panels show PHEV infected cells double stained for PHEV (green) and cytokeratin 18 (CK18), a marker of sustentacular cells (red); nuclei in blue. PHEV infected cells are double positive for CK18 at all collected time points Scale bar = 50 µm **B** Quantification of the number of infected cells per 10 sections (magnification 400×). **C** Extracellular virus titers. Values represent mean ± standard deviation (SD) from independent experiments from three animals. Significant differences between the different conditions (One-way ANOVA df = 3, F = 69.616 at 24 hpi; df = 3, F = 41.214 at 48 hpi followed by Tukey’s post-hoc tests) are visualized using CLD. Significant differences are indicated by letter annotations, where lowercase letters correspond to 24 hpi and uppercase letters correspond to 48 hpi. Groups not sharing the same letter are significantly different (*p* < 0.05).

To determine whether the historical and contemporary strains of PHEV differ in their replication kinetics and cell tropism in the host olfactory mucosa, infection was evaluated in olfactory mucosa explants with and without prior disruption of tight junctions. The infection patterns observed in olfactory mucosa explants revealed clear differences between the historical VW572 strain and the contemporary PS412 strain of PHEV using a double immunofluorescence staining of olfactory mucosal explants using a sustentacular cell-specific marker (CK18) in combination with viral antigen detection (Figure 4A). All PHEV positive cells were CK18 double positive, demonstrating that only the sustentacular cells were susceptible to PHEV infection within the olfactory epithelium. VW572 infected more sustentacular cells at 24 hpi and 48 hpi than PS412. EGTA treatment had a significantly increasing effect on the number of VW572 infected cells at 24 hpi (Figure 4B, *p* < 0.001); it had no effect on the number of PS412 infected cells. Interestingly, the extracellular virus titers did not follow the pattern of the number of infected cells. PS412 produced higher virus titers than VW572, indicating greater productivity in infected olfactory epithelial cells. At 24 hpi and 48 hpi, PS412 was respectively 1.19-fold and 1.64-fold more productive than VW572. Collectively, PHEV infection in olfactory mucosa is restricted to sustentacular cells, with VW572 infecting more cells but PS412 achieving higher viral productivity, indicating strain-specific differences in infection dynamics.

### Historical VW572 strain shows significantly higher EGTA driven infection in PoRECs

**Figure 5:**
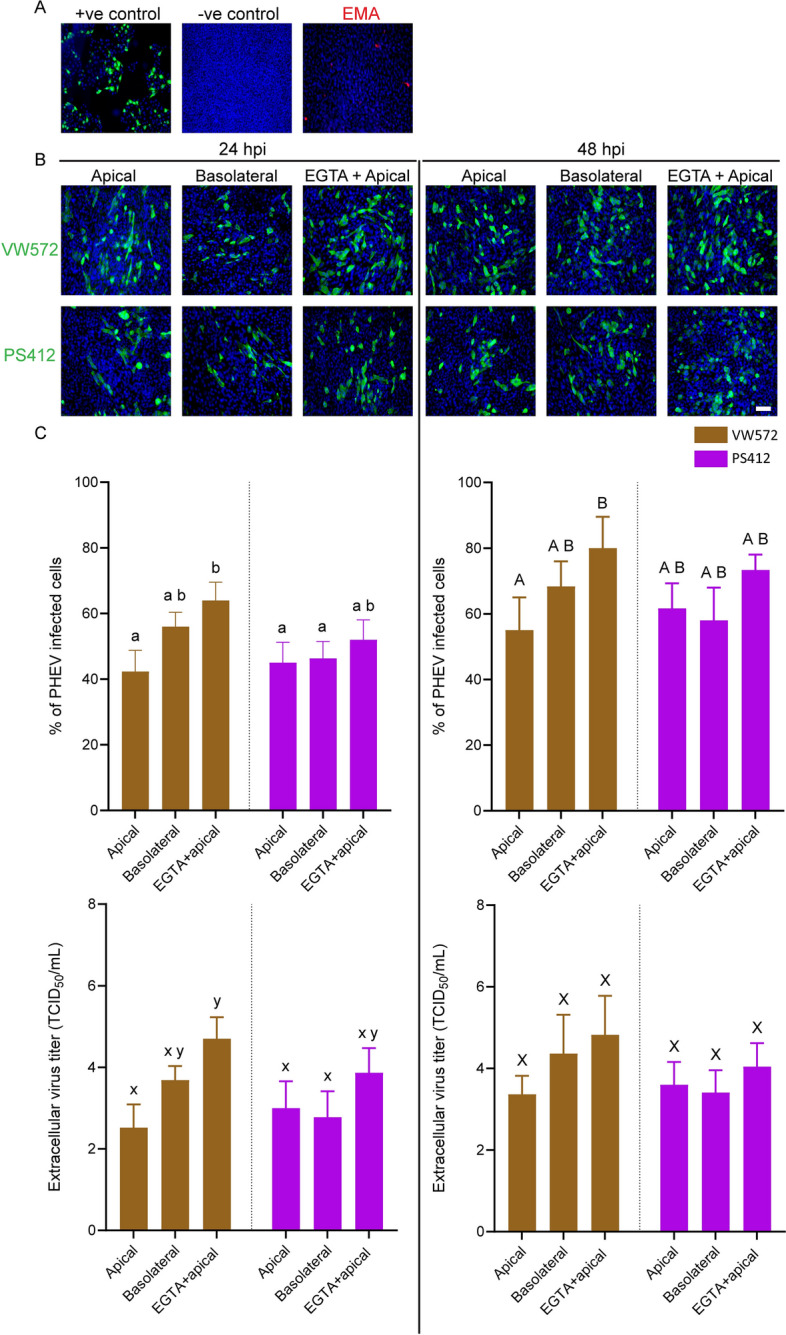
**PHEV VW572 and PS412 replication in PoRECs (n = 3).** **A** Positive control: Virus-inoculated RPD cells showing characteristic cytoplasmic staining. Negative control: Mock-inoculated porcine respiratory epithelial cells (PoRECs). Cell viability in mock-inoculated PoRECs was confirmed using EMA (ethidium monoazide) staining. Red cells are non-viable cells. Scale bar = 50 µm **B** Representative images of PHEV infected cells in PoRECs when infected apically (with and without EGTA treatment) or basolaterally. PHEV-infected cells are green; nuclei are blue (magnification 400×). **C** The percentage of PHEV infection for both strains of PHEV at 24 hpi and 48 hpi **D** Extracellular virus titer of PoRECs at 24 hpi and 48 hpi for both strains of PHEV. Values represent mean ± SD from independent experiments from three animals. Significant differences between the different conditions (One-way ANOVA VW572: df = 2, F = 11.828 at 24 hpi; df = 2, F = 5.648 at 48 hpi; PS412: df = 2, F = 1.215 at 24 hpi; df = 2, F = 3.194 at 48 hpi followed by Tukey’s post-hoc tests) are visualized using CLD. Significant differences are indicated by letter annotations, where lowercase letters correspond to 24 hpi and uppercase letters correspond to 48 hpi. Groups not sharing the same letter are significantly different (*p* < 0.05).

To explore whether epithelial polarity differentially affects infection by both PHEV strains, polarized primary porcine respiratory epithelial cell (PoRECs) were used. PoRECs were highly viable (> 98.99%; 95% CI 98.00–99.98%) after attaining confluency, as confirmed by EMA-stained images (Figure 5A). PHEV VW572 and PS412 infected similar numbers of cells in PoRECs through both apical and basolateral infection routes (Figure 5B). The confluency of the epithelial cultures was verified by measuring transepithelial electrical resistance (TEER). EGTA treatment increased the replication of both strains; however, it was statistically significant for only VW572 (*p* = 0.0009) (Figure 5C). Under standard apical infection conditions, PS412 showed a numerically higher level of infection than VW572, but this difference was not statistically significant. Following EGTA treatment, VW572 exhibited a significant enhancement in infection, whereas PS412 showed a mild, non-significant trend toward increased infection. These results suggest that the two strains differ in their response to epithelial junction disruption.

### RBD mutations in both strains may alter their structure and function

**Figure 6:**
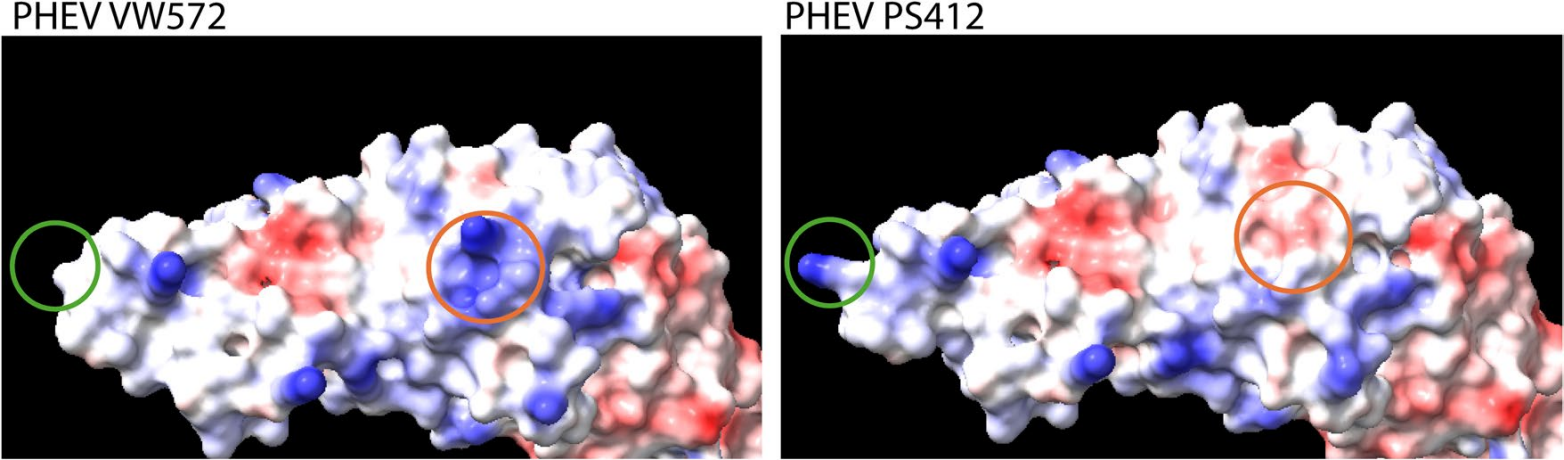
**3D structure of the receptor binding site of the spike protein of both PHEV strains.** In these representations, blue regions denote positively charged areas, red regions indicate negatively charged areas, and white regions correspond to neutral/polar uncharged/hydrophobic surfaces. Green circles highlight structural and electrostatic changes associated with a point mutation at amino acid position 512; orange circles indicate alterations resulting from a point mutation at position 576.

To identify potential genetic differences that could underline the observed phenotypic variation between the historical VW572 and contemporary PS412 strain of PHEV, whole-genome sequences were compared and structural modeling of the spike protein was performed. The genome of PS412 (30431 nucleotides) was slightly shorter than that of VW572 (30467 nucleotides). 3D structures of the spike proteins and RBD of both strains were predicted based on AlphaFold 3 software and visualized by Chimera X (Figure 6 and Additional file 1). In the deduced amino acid sequence, there were seven amino acid mutations. Two of them were in the predicted receptor binding domain of the S1 protein, at positions 512 and 576 [7, 29]. At position 512, the amino acid was asparagine (polar, uncharged) for VW572 and lysine (positively charged) for PS412. At that position, the spike protein of the PHEV PS412 strain had a small protrusion which was positively charged, while it was absent in the VW572 strain. At position 576, VW572 had a lysine (positively charged) while PS412 had a glutamic acid (negatively charged). These genomic differences in the receptor binding domain of both strains may influence their structural and functional properties.

## Discussion

PHEV is known to cause two distinct clinical syndromes in swine, vomiting and wasting disease (VWD) and encephalomyelitis. VWD is characterized by persistent vomiting, dehydration, and progressive weight loss, while encephalomyelitis presents with neurological signs such as ataxia, hyperesthesia, incoordination, tremors, and paddling movements, often progressing to recumbency and death in severe cases. In addition to its neurotropism, previous studies have demonstrated that PHEV can replicate in the tracheal and bronchial epithelial cells, indicating a potential involvement in respiratory disease [30]. VW572 was isolated in our laboratory in 1972 [11]. It mainly exhibited a strong neurotropism particularly in young piglets [31]. Over time, the pathogenesis (tissue tropism) and clinical signs of betacoronavirus infections may have changed due to genetic mutations [32, 33]. In a recent study, it was shown that currently circulating PHEV strains cause more flu-like symptoms [34]. Nasal respiratory epithelial cells are the primary replication site of PHEV infection as shown by other studies [1, 35]. Olfactory epithelial cells may also be infected by PHEV [19]. Hence, we decided to compare the replication behavior of the historical VW572 and contemporary PS412 strains, which was isolated from nasal swabs during a monitoring study, in the fully susceptible RPD cell line, porcine respiratory and olfactory explants, and PoRECs.

Epithelial cells exhibit apical and basolateral receptors, which play a vital role in viral infection by mediating entry, replication and spread [36–38]. These receptors are separated by tight intercellular junctions (ICJs) [39]. These junctions may affect viral tropism and pathogenesis. Some viruses favor attachment at the apical side like respiratory syncytial virus, while viruses like adenoviruses and alphaherpesviruses show a preference for the basolateral side [40, 41]. The integrity of ICJs is critical for maintaining this polarity and disruption of ICJs can expose previously inaccessible receptors, potentially altering infection patterns and pathology/disease [39]. EGTA is a calcium chelator that disrupts ICJs in epithelial tissues, which is reversible, and the epithelium itself repairs within 24 h after EGTA removal [42]. Recent research from our lab demonstrated that different environmental nuisances such as pollens, spores and bacterial proteases can destroy or morphologically change the ICJs, allowing viruses to reach basolaterally exposed receptors [43, 44]. In this study, we checked the effect of EGTA on both explants and PoRECs. The exposure of basolateral receptors by disrupting the ICJs using EGTA revealed clear patterns of viral tropism for the two PHEV strains across different respiratory tissues. In our study, the absence of a significant increase in viral replication upon EGTA treatment in nasal explants for both strains suggests that viral entry likely occurs primarily through apical surface receptors. This interpretation is consistent with the presumed in vivo situation, where infection initiates at the mucosal surface of the nasal epithelium. Interestingly, a marked enhancement in infection was observed in PoRECs following EGTA pretreatment for VW572; this was not the case for PS412 strain. These findings imply possible differences in receptor accessibility for both strains, and physiological differences between ex vivo explants and in vitro cultured epithelial cells.

In the olfactory mucosa explant model, a significant difference in infection levels was observed between the two PHEV strains, with the VW572 strain demonstrating markedly higher number of infected cells. Pretreatment with EGTA resulted in a marked increase in susceptibility to infection by the VW572 strain. This finding indicates that disruption of the epithelial barrier significantly enhances viral access to permissive cellular targets within the olfactory mucosa. Although we did not directly assess receptor localization following EGTA treatment in this study, our interpretation is supported by previous work from our laboratory, which demonstrated that EGTA treatment can expose basolaterally located viral receptors in the respiratory epithelium by disrupting intercellular junctions [44]. Double immunofluorescence labeling demonstrated the co-localization of viral antigens with a marker specific for sustentacular cells, providing robust evidence that these supporting cells constitute a primary target population for PHEV during early stages of infection [45]. Sustentacular cell dysfunction disrupts olfactory epithelial integrity, impairing extracellular ion balance and epithelial barrier function, which may permit viral particles or inflammatory mediators to reach the olfactory bulb [46, 47]. While the VW572 strain’s sustentacular tropism may enhance neuroinvasion risk through these routes, the PS412 strain’s reduced mucosal infectivity, suggesting an attenuated neurotropic potential, possibly due to receptor-binding or replication adaptations favoring respiratory over neural tissues. These findings underscore the role of sustentacular cells as gatekeepers of olfactory barrier function and highlight how viral evolution may alter neuroinvasion strategies. In addition, we attempted to identify olfactory neurons using commonly used neuronal markers, including neuronal cell adhesion molecule (CD56/NCAM), olfactory marker protein (OMP, Invitrogen), and β-tubulin III, to differentiate further PHEV susceptible cells in the olfactory mucosa. However, none of these markers showed a labeling of olfactory neurons in porcine olfactory epithelium due to lack of cross reactivity for porcine olfactory neurons. This represents a methodological limitation of our study.

Nasal mucosal explants preserve the native 3-dimensional tissue architecture, including an intact basement membrane, fully differentiated epithelial and submucosal cell layers. Moreover, in the explant model, mucociliary clearance and other innate defense mechanisms may further limit viral access even when epithelial integrity is chemically challenged. In contrast, differentiated PoRECs, while capable of forming tight junctions and cilia in vitro, may exhibit increased susceptibility to EGTA induced junctional disruption, resulting in greater exposure of basolateral receptors that facilitate viral entry. It is very well possible that by enzymatic treatment of the nasal mucosa tissues for releasing the epithelial cells and subsequent cultivation of these cells, receptors may no longer be expressed or become redistributed. PS412 and VW572 replicated in a similar way in the nasal mucosa. Thus, the replication in the nasal mucosa does not explain the enhanced neuroinvasive capacity for VW572.

Taking the nasal and olfactory infection models in consideration, the olfactory route seems to be an important entry site for PHEV to invade neural tissues. It highlights the existence of strain differences in mucosal entry and subsequent dissemination strategies, underlining the need for further mechanistic studies to delineate the relationship between mucosal replication dynamics and central nervous system invasion. A recent study also reported strain specific differences in neurotropism of murine neuronal cell line between VW572 and PS412, where VW572 displayed a stronger neuronal infection than PS412 [21]. From these studies in mice and pigs, it may be speculated that PHEV originally came from rodents and entered the pig population in the seventies (reference strain VW572); afterwards, the virus may have adapted to pigs with a reduced replication in the ethmoidal mucosa.

Ex vivo studies with respiratory and olfactory explants provide a bridge between in vitro and in vivo models for investigating the first stages of viral replication. The explants preserve the cellular diversity and complex tissue architecture and mimic the natural infection site [48]. By using PoRECs and respiratory and olfactory explants, we specifically focused on the PHEV-respiratory epithelial cell interactions [49]. To ensure the validity of our findings regarding PHEV infection and replication kinetics, viability assessment had to be performed for both explants and PoRECs. We used a TUNEL assay for explants to detect apoptotic cells and observed only limited apoptosis throughout the experimental period, suggesting that the tissue structure and cellular integrity were largely maintained. However, since TUNEL detects only apoptotic cell death, it does not fully represent total cell viability. An EMA staining was used for analyzing the viability in PoRECs. These cell cultures remained highly viable over time. This approach ensured that the observed replication kinetics of both PHEV strains in both explants and cell cultures were based on interactions with viable, fully functional respiratory epithelial tissues and cells. Our methodology, utilizing both tissue explants and PoRECs provides a robust and comprehensive approach to study PHEV infection behavior across different strains.

Our study identified two amino acid changes in the receptor-binding domain of the S protein between the historical and contemporary PHEV strains. Due to these changes, a clear charge shift occurred at two positions in the spike protein of PHEV. These charge changes in the spike protein may affect the virus-host interaction, viral entry and immune evasion [50, 51]. Recently, key amino acids in the PHEV spike protein that interact with the DPP1 receptor in swine kidney cells were identified with positions 505, 507, 510, 511, 514, and 533 being critical for receptor binding [22]. In our study, the residues highlighted as potentially important were 512 and 576, all located within the receptor binding domain. The differences in amino acid positions between the two studies may reflect biological and experimental variations. The reference study was performed using a swine kidney cell line, whereas our focus was on primary respiratory tissues, which may influence virus host interactions. Furthermore, the strain used in the previous study was 67 N, while our study investigated the PS412 and VW572 strains. These differences suggest that PHEV may exhibit strain specific interactions within different host tissues, and it is also possible that additional or alternative receptors in respiratory tissues contribute to viral entry and tropism. Despite these spike differences, replication kinetics in nasal explants showed no significant variation post EGTA treatment, indicating that both strains use similar entry mechanisms and replication strategies in the nasal epithelium. However, VW572 replicated better in olfactory mucosa explants than PS412 and EGTA had only an impact for VW572 in olfactory mucosa explants. Taking these data together, it can be concluded that PHEV PS412 lost some of its capacity to replicate in the olfactory mucosa. Though, despite the lower number of infected cells, the production per infected cell was higher than for VW572 (1.64×). We can also conclude that VW572 uses (a) basolateral receptor(s) in the olfactory mucosa and PoRECs, while PS412 strain favors apical receptors. This is indicative for the use of different receptors in the olfactory mucosa for the two strains. Although AlphaFold 3 can predict the structures of glycosylated proteins, the models remain computational predictions. Experimental techniques such as cryo-electron microscopy (cryo-EM) remain the gold standard for determining the precise conformation of spike glycoproteins.

This study primarily used ex vivo explant models, which are more representative than cell cultures but may not fully mimic the in vivo situation. Future studies should include in vivo experiments, a broader range of PHEV strains, and detailed molecular analyses of the viral ligands and host receptors to enhance our understanding of PHEV pathogenesis and strain-specific behaviors.

This study serves as a baseline comparison of replication kinetics and epithelial tropism between two PHEV strains. While mechanistic assays were not included, our aim was to establish foundational infection patterns using ex vivo respiratory explants. Future studies will build on these findings to explore molecular mechanisms in greater depth.

## Supplementary Information

 **Additional file 1** **Full spike protein structure of both PHEV strains (VW572 and PS412).** Blue regions denote positively charged areas, red regions indicate negatively charged areas, and white regions correspond to neutral/polar uncharged/hydrophobic surfaces. Structures were predicted based on AlphaFold software and visualized by Chimera X.

## Data Availability

All relevant data has been included in the main text. Unpublished data are available from the corresponding author on reasonable request.
