# Supplementary material for: Different replication behavior of a contemporary porcine hemagglutinating encephalomyelitis virus strain Gent/PS412 compared with the historical neurotropic reference strain VW572
Source: Vet Res. 2026 Jun 24;57:115. doi: 10.1186/s13567-026-01767-1 (PMC13295890; doi:10.1186/s13567-026-01767-1)
Supplement: Supplementary file 1 — Additional file 1 Full spike protein structure of both PHEV strains (VW572 and PS412). Blue regions denote positively charged areas, red regions indicate negatively charged areas, and white regions correspond to neutral/polar uncharged/hydrophobic surfaces. Structures were predicted based on AlphaFold software and visualized by Chimera X. [file 13567_2026_1767_MOESM1_ESM.pdf]

Additional file 1:

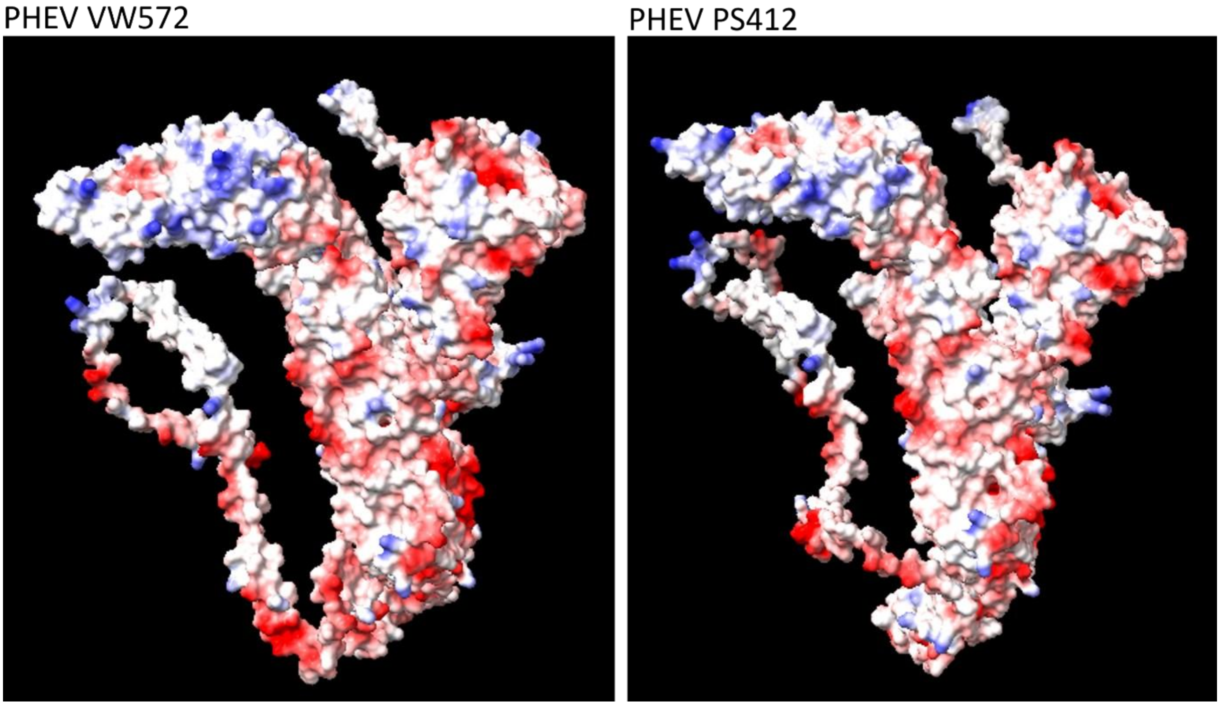

Additional Figure 1. Full spike protein structure of both PHEV strains (VW572 and PS412). Blue regions denote positively charged areas, red regions indicate negatively charged areas, and white regions correspond to neutral/polar uncharged/hydrophobic surfaces. Structures were predicted based on AlphaFold software and visualized by Chimera X.
